# 

**Published:** 1885-10

**Authors:** 


PAGE.

ORIGINAL COMMUNICATIONS.

Congenital Paralysis of the Oculo-Motor of Both Sides. Tilley.... 305

Five Cases of Ovariotomy. C. G. Davis.... 312

HOSPITAL REPORTS.

*Cook County Hospital.* Forty-five Cases of Typhoid Fever. F. S. Johnson.... 328

*St. Luke's Hospital.* Multiple Abscess of the Liver. Cary.... 340

EDITORIAL.

The Ninth International Medical Congress.... 342

Reports of the Transactions of the Chicago Medical Societies.... 344

DOMESTIC CORRESPONDENCE.

A Primer Lesson in "Metaphysics" 347

A Case of Poisoning by Muriate of Cocaine.... 349

SOCIETY REPORTS.

*The Chicago Gynecological Society, Meeting of July 17th.* Considerations Relating to Shock and Nervous Influence in Parturition. Newman.... 351

Diet and Management of Artificially Fed Infants. Foster.... 352

*The Chicago Gynecological Society, Meeting of September 18th.* Abdominal and Gynecological Surgery in England, Scotland and Heidelberg. Dudley.... 356

PAGE.

*Chicago Medical Society, Meeting of September 21st.* Special *versus* General Study in Medicine. Sinclair.... 367

Treatment of Syphilis. Potter.... 369

Retention of Sponge-Tent in Cervix Uteri. Parkes.... 373

American Dermatological Association.... 374

Report of the Committee Appointed to Arrange for the Meeting of the International Medical Congress in America in 1887.... 379

The First Meeting of the Executive Committee of the Ninth International Medical Congress.... 391

BOOK REVIEWS.

A Guide to Diseases of Children. Goodhart.... 393

A System of Oral Surgery, etc. Garretson.... 395

Manual for Hospital Nurses. Domville.... 396

An Introduction to the Study of Diseases of the Nervous System. Stewart.... 397

Urinary and Renal Derangements. Beale.... 398

OBITUARY.

Dr. P. A. Allaire, of Aurora, Ill.... 399

NEWS ITEMS.... 399

EXTRACTS AND ABSTRACTS.... 400

